# A Gravity-Driven Membrane Bioreactor in Treating Real Fruit Juice Wastewater: Response Relationship Between Filtration Behavior and Microbial Community Evolution

**DOI:** 10.3390/membranes14120260

**Published:** 2024-12-06

**Authors:** Dan Song, Haiyao Du, Shichun Chen, Xiaodie Han, Lu Wang, Yonggang Li, Caihong Liu, Wenjuan Zhang, Jun Ma

**Affiliations:** 1 School of Marine Science and Technology, Harbin Institute of Technology at Weihai, Weihai 264209, China; 2 PetroChina Harbin Petrochemical Company, Harbin 150056, China; 3 Guangxi Key Laboratory of Urban Water Environment, Baise University, Baise 533000, China; 4 Key Laboratory of Eco-Environments in Three Gorges Reservoir Region, Ministry of Education, College of Environment and Ecology, Chongqing University, Chongqing 400044, China; 5 Tianjin Key Laboratory of Aquatic Science and Technology, School of Environmental and Municipal Engineering, Tianjin Chengjian University, Tianjin 300384, China

**Keywords:** gravity-driven membrane bioreactor (GDMBR), fruit juice wastewater treatment, sludge characteristics, biofilm, microbial community

## Abstract

The issue of environmental pollution caused by wastewater discharge from fruit juice production has attracted increasing attention. However, the cost-effectiveness of conventional treatment technology remains insufficient. In this study, a gravity-driven membrane bioreactor (GDMBR) was developed to treat real fruit juice wastewater from secondary sedimentation at pressures ranging from 0.01 to 0.04 MPa without requiring backwashing or chemical cleaning, with the aim of investigating flux development and contaminant removal under low-energy conditions. The results demonstrate an initial decrease in flux followed by stabilization during long-term filtration. Moreover, the stabilized flux level achieved with the GDMBR at pressures of 0.01 and 0.02 MPa was observed to surpass that obtained at 0.04 MPa, ranging from 4 to 4.5 L/m^−2^ h^−1^. The stability of flux was positively associated with the low membrane fouling resistance observed in the GDMBR system. Additionally, the GDMBR system provided remarkable efficiencies in removing the chemical oxygen demand (COD), biological oxygen demand (BOD), ammonia (NH_4_^+^-N), and total nitrogen (TN), with average removal rates of 82%, 80%, 83%, and 79%, respectively. The high biological activity and microbial community diversity within the sludge and biofilm are expected to enhance its biodegradation potential, thereby contributing to the efficient removal of contaminants. Notably, a portion of total phosphorus (TP) can be effectively retained in the reactor, which highlighted the promising application of the GDMBR process for actual fruit juice wastewater based on these findings.

## 1. Introduction

The wastewater generated during juice production typically exhibits characteristics such as elevated viscosity, substantial levels of chemical oxygen demand (COD) and biological oxygen demand (BOD), and significant quantities of suspended solid impurities (SS) [1,2]. If discharged directly into water bodies without appropriate treatment, it can significantly impact the aquatic ecosystem, leading to the severe degradation of the surrounding water quality [3], the disruption of the ecosystem structure [4], and frequent occurrences of fish and shrimp poisoning [5], diseases, or mortality [6]. Moreover, the nearby crop productivity and quality are adversely affected. Therefore, achieving an efficient removal of these contaminants remains a pressing challenge that must be addressed in the current treatment of fruit juice wastewater.

The membrane bioreactor (MBR) technology, known for its exceptional rejection capability, compact footprint, effortless automatic operation and control, and modular design, has been extensively investigated and applied in the realm of wastewater treatment [7]. The MBR process can effectively combine biological degradation with membrane retention, resulting in the production of high-quality permeate while minimizing space requirements [8]. Additionally, this integration also promotes biomass retention and prevents the efflux of suspended microorganisms [9]. However, due to the formation of severe membrane fouling, the conventional MBR technology requires periodic backflushing and chemical cleaning, thereby resulting in increased operational and maintenance costs, energy consumption, and chemical usage [10]. These limitations impede its further application. Hence, exploring strategies for minimizing energy consumption and optimizing the operation and maintenance of MBR technology has emerged as a novel research focus in the wastewater treatment field.

The gravity-driven membrane bioreactor (GDMBR) is extensively employed as a representative ultra-low pressure membrane filtration mode in the treatment of domestic wastewater, secondary effluent [7,11], seawater [12,13], and shale gas wastewater [14]. On the one hand, the GDMBR still combines the degradation of biofilms with membrane rejection, retaining the inherent advantages of the MBR process [15,16]. On the other hand, operating under extremely low gravitational pressure conditions (0.2–0.7 m), the GDMBR process eliminates the need for physical or chemical cleanings and effectively addresses the drawbacks associated with high energy consumption and frequent backwashing in conventional MBR technology [17,18]. According to previous studies, the GDMBR has a stable flux, which is attributed to the formation of heterogeneous, rugged, and porous biofilms on the membrane surface [19]. It also demonstrated that the heterogeneity and permeability of biofilms can be augmented through intensified biological predation within the biofilms, resulting in an elevated level of steady membrane flux [20,21]. Additionally, the formation of biofilms also enhances the GDMBR’s efficiency in effectively removing colloids, suspended materials, microorganisms, organic compounds, ammonia, iron, manganese, etc. [7,22]. Meanwhile, the biofilm acts as a pre-filter to also mitigate direct contact or deposition between contaminants and membrane pores [23]. To further enhance the filtration performance of the GDMBR, auxiliary materials such as granular activated carbon (GAC) are commonly incorporated, resulting in a significant improvement in the stable flux levels and removal efficiencies of dissolved organic carbon (DOC), ammonia, etc. [23,24]. At present, the GDMBR has been utilized for grey water and domestic wastewater treatment, and the findings indicate that the flux remains stable, albeit at relatively low levels of 1–2 L m^−2^ h^−1^ [25,26]. Furthermore, it was observed that the characteristics of the influent water, particularly the concentrations of organic substances, exhibit a direct correlation with both the flux stabilization and achieving a steady-state flux level [27,28]. In the fruit juice wastewater, further investigation is required to achieve flux stabilization and contaminant removal due to the significantly higher concentration of organic contaminants characterized by COD and BOD compared to those reported in previous studies on the GDMBR. To the best of our knowledge, there is a limited number of reports available on the treatment of fruit juice wastewater using GDMBR technology. However, most of these studies have utilized various filters or filter layers such as GAC, biocarrier, synthetic fiber, lava stone, and sand. The incorporation of such additional components may potentially lead to increased capital costs, larger footprint sizes, and higher operation and maintenance requirements while diminishing the inherent advantages offered by GDMBR technology. Therefore, it is crucial to determine whether the GDMBR process can effectively treat fruit juice wastewater without requiring direct integration facilities or pre-treatments, while also evaluating the impact of microbial community evolution on its filtration performance.

Therefore, the objective of this study was to employ the GDMBR process for the direct treatment of fruit juice wastewater. The investigation focused on optimizing the process, achieving a stabilizing flux level, and evaluating the removal performance of various contaminants during GDMBR filtration. In order to gain insights into the fouling behavior and mechanisms responsible for flux stability in the GDMBR system, we conducted an examination of sludge size changes, internal structures, and the microbial community composition of sludge and biofilm adhered on the membrane surface. Additionally, we assessed how different microbial communities impact the performance of the GDMBR process in treating fruit juice wastewater.

## 2. Materials and Methods

### 2.1. Fruit Juice Wastewater

The influent was collected from the fruit juice wastewater of Yantai North Andli juice Co., Ltd., Shandong, China. The characteristics of wastewater were monitored to be as follows: a pH of 4.22–7.01, an electric conductivity of 1882.25–1918.12 us/cm, an SS of 134.68–233.23 mg/L, an NH_4_^+^-N of 61.71–140.18 mg/L, a COD of 399.48–869.75 mg/L, a BOD of 160.71–460.58 mg/L, total nitrogen (TN) of 170.21–325.41 mg/L, total phosphate (TP) of 4.39–10.98 mg/L, and a Cl^−^ of 103 mg/kg. The data were recorded by averaging triplicate tests.

### 2.2. Experimental Setup and Operating Conditions

**Figure 1:**
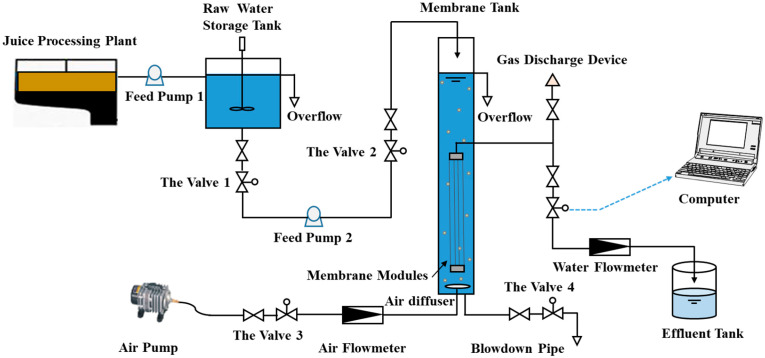
Schematic diagram of the GDMBR system in treating fruit juice wastewater directly.

A schematic diagram illustrating the configuration of our GDMBR system is presented in Figure 1. The fruit juice wastewater was pumped into a 100 L plexiglass vessel from the top and recirculated through an overflow pipe to maintain a constant driving force. The vertically oriented membrane module operated in submerged filtration mode, with continuous air aeration at a rate of 10 L min^−1^ to scour the membrane surfaces and provide essential dissolved oxygen levels (6.5 mg L^−1^). The hydraulic retention time was approximately 15 h, and activated sludge obtained from biological wastewater treatment plants was introduced without undergoing any sludge discharge or membrane cleaning procedures during operation. To ensure consistent experimental conditions, the operational temperature remained at 25 ± 1 °C for approximately two months. The experimental membranes utilized were hollow fiber polyvinylidene fluoride ultrafiltration membranes with pore sizes ranging from 0.05 to 0.3 μm provided by the Sino-Euro Institute of Membrane Technology, China; water fluxes were accurately measured using electronic balances and recorded on a computer system. The membrane fouling resistance was calculated according Text S1 in the Supporting Information (SI).

### 2.3. Analytical Methods

#### 2.3.1. Microbial Community Analytical Methods

The DNA extraction was performed using the E.Z.N.A.Soil DNA Kit (OMEGA, Norcross, GA, USA), with 0.2 g of each sample from raw water, sludge, and biofilm. Subsequently, the concentration and integrity of the extracted DNA were assessed using a Qubit 2.0 fluorimeter (Invitrogen, Carlsbad, USA) and gel electrophoresis system (Analytic Jena, Jena, Germany). The yield and purity of the DNA were evaluated for polymerase chain reaction (PCR) using the Qubit 2.0 DNA kit (Life Technologies, Carlsbad, CA, USA). For PCR amplification targeting the V3–V4 region of interest, primers 341F (CCTACACGACGCTCTTCCGATCTN (barcode) CCTACGGGNGGCWGCAG) and 805R(GACTGGAGTTCCTTGGCACCCGAGAATTCCAGACTACHVGGGTATCTAATCC) were employed. Illumina bridge-compatible PCR primers were subsequently utilized with barcodes added to the 5 end of both the 349F and 341F primers to enable multiplexing. The raw reads with a length of less than 50 bp were filtered out using Ureseach 5.2.236 [29] in the data sets. A total of more than 10,000 sequences were obtained from the samples. Clustering was performed based on sequence distances, and operational taxonomic units (OTUs) were defined at a similarity threshold of 99%. OTU assignments were made by matching against the RDP database (RDP classifier 2.10.1), and clustering was conducted using uclust (uclust v1.1.579). The Shannon diversity index was calculated using mother (mother 1.30.1), and sequence processing and analysis utilized the Silva database [30].

#### 2.3.2. Water Quality Analytical Methods

COD, BOD, SS, NH_4_^+^-N, TP, mixed liquor suspension solids (MLSS), and mixed liquor volatile suspension solids (MLVSS) as well as the sludge volume index (SVI) were measured according to standard methods that are presented in Texts S2 in SI [31]. The sludge particle sizes were investigated using a laser particle size analyzer (Malvern model, Malvern ZS90). Total nitrogen (TN) was determined using a TOC analyzer (multi N/C 2100S, Analytic Jena, Germany). Bacterial counts of the reactor and effluent were measured by Accuri C6 Flow Cytometry (BD, New Jersey, USA). The DO of liquids was monitored using an HQ30d meter (HACH, Loveland, CO, USA). The concentrations of metal ions were detected by Inductively Coupled Plasma-Mass Spectrometry (ICP-MS) (PerkinElmer, Waltham, MA, USA). The deionized water was recorded as the blank. The temperature of the system was at room temperature (25 ± 1 °C).

## 3. Results and Discussion

### 3.1. Flux Variation and Filtration Resistance Distribution

**Figure 2:**
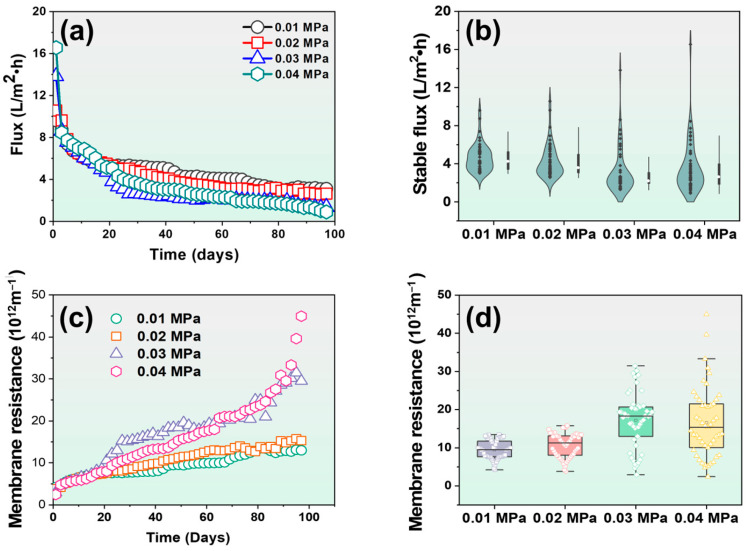
(**a**) Variation in water flux with operation time; (**b**) stable flux; (**c**) variation in membrane resistances with operation time; and (**d**) stable membrane resistance during the operation.

The temporal dynamics of water flux and the distribution of resistance in GDMBR systems were systematically assessed. The membrane flux variations and stable fluxes in the GDMBR under different pressures (0.01, 0.02, 0.03, and 0.04 MPa) during long-term treatments of fruit juice wastewater are illustrated in Figure 2a,b. According to the membrane flux curve, even with an increase in driving pressure from 0.01 to 0.04 MPa, the decline in flux exhibits a consistent trend, which can be roughly categorized into two distinct stages. In the initial stage (0–20 days), a significant decline in flux was observed, with values decreasing from 9.61 L m^−2^ h^−1^, 10.57 L m^−2^ h^−1^, 13.83 L m^−2^ h^−1^, and 16.55 L m^−2^ h^−1^ to 5.37 L m^−2^ h^−1^, 5.48 L m^−2^ h^−1^, 4.73 L m^−2^ h^−1^, and 5.14 L m^−2^ h^−1^, respectively, resulting in flux decline rates of 44.12%, 48.16%, 65.79%, and 68.94%, respectively. Despite the varying pressures applied to the four GDMBR systems, their membrane fluxes exhibit remarkable similarity after a duration of 20 days. Additionally, the membrane fluxes of the GDMBR under 0.04 MPa and 0.03 MPa exhibited significantly higher values compared to the other two GDMBRs on the initial day due to their elevated membrane driving pressures; however, as filtration progressed, their membrane fluxes gradually converged. The occurrence of this phenomenon can be attributed to the relatively high concentrations of particles, suspended substances, and colloids present in the fruit juice wastewater that were rejected on the membrane surface, resulting in the rapid formation of a cake layer. Consequently, a significant acceleration in membrane flux decline was observed. Noteworthily, the initial flux level of the GDMBR systems exhibited a notable decrease compared to previous studies, likely attributed to the presence of a higher concentration of organic contamination in the feed water [25,32,33].

The fluxes of all of the GDMBRs exhibited a tendency to attain a state of equilibrium during the period from day 21 to day 97. The stable fluxes of the GDMBR under 0.01, 0.02, 0.03, and 0.04 MPa were measured as 4.28 L m^−2^ h^−1^, 3.51 L m^−2^ h^−1^, 2.18 L m^−2^ h^−1^, and 2.56 L m^−2^ h^−1^, respectively. Despite the variation in driving pressure among the GDMBR groups, their stable flux levels exhibited similarity, suggesting that the driving pressure exerted a negligible influence on the stable flux of the GDMBR process for treating fruit juice wastewater. Previous studies have also indicated a slightly negative impact of the driving pressure on the stabilized fluxes of the GDMBR systems; higher driving pressures were associated with a reduced stable flux [34,35]. The potential explanation was that an increased driving pressure led to a denser biofilm adhering to the membrane surface, resulting in more severe membrane fouling. In particular, the high driving pressure during the initial filtration stage led to increased water penetration through the GDMBR, resulting in an enhanced contact and accumulation of foulants within the membrane pores, thereby causing severe pore blocking. Reinforcing this perspective, prior research has also demonstrated that biofilms developed on the surface of ultrafiltration membranes under lower driving pressures exhibit a higher likelihood of being physically dislodged compared to those formed under higher driving pressures [36]. Therefore, in the long-term filtration of the GDMBR, better permeability was observed at 0.01 and 0.02 MPa compared to 0.03 and 0.04 MPa (Figure 2b). The concentration of organic contaminants, such as TOC, played a pivotal role in the development and stabilization of flux during long-term GDMBR filtration. A higher TOC concentration resulted in a lower attainable stable flux [37].

The variations in the membrane fouling resistances of the GDMBR systems during the long-term operations are illustrated in Figure 2c,d. The development of membrane fouling resistance demonstrated a strong inverse correlation with variations in water flux. The GDMBR systems operated at 0.03 and 0.04 MPa consistently exhibited a higher rate of membrane fouling resistance growth throughout the entire experimental period. The increases in membrane fouling resistance were relatively slow when the GDMBR system operated at 0.01 and 0.02 MPa, suggesting that lower driving pressures effectively enhanced the anti-fouling performance of the GDMBR, with a more pronounced effect observed at lower pressures. The distributions of stable fouling resistances on the membrane in four GDMBR systems were further analyzed (Figure 2d). The GDMBR systems operated at pressures of 0.01 and 0.02 MPa exhibited significantly lower membrane fouling resistances, measuring 10.02 × 10^12^ m^−1^ and 10.78 × 10^12^ m^−1^, respectively. It is worth noting that the GDMBR systems operated at pressures of 0.03 and 0.04 MPa showed higher membrane fouling resistances, measuring 16.99 × 10^12^ m^−1^ and 16.48 × 10^12^ m^−1^, respectively, which represents a noticeable increase compared to those observed in the GDMBR system operated at pressures of 0.01 and 0.02 MPa. The observed discrepancy can be ascribed to the presence of a higher concentration of dissolved organic matter in the feed water, thereby enhancing the likelihood of pore blockage under elevated operating pressures [38]. Furthermore, as the operation time prolongs, the absence of microbial movement might lead to a more dense fouling layer on the membrane surface [39]. Therefore, the system operating pressure of the subsequent experiment was set at 0.01MPa.

### 3.2. Permeate Quality of Gravity-Driven Membrane Bioreactor System

#### 3.2.1. Removals of Organic Substances

**Figure 3:**
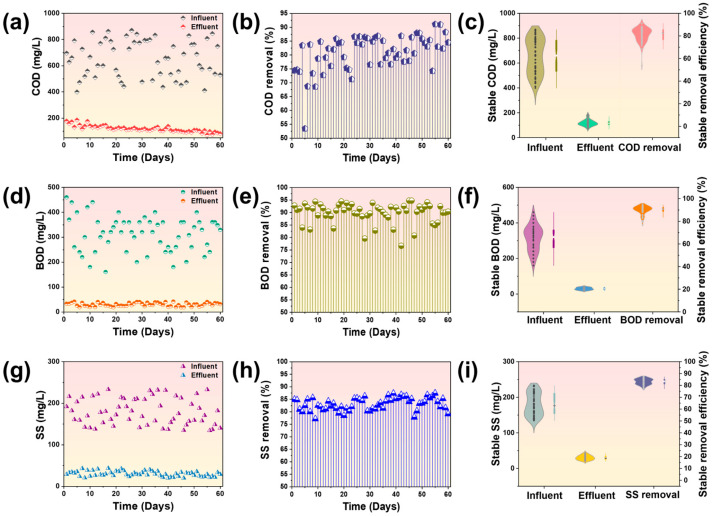
(**a**) COD concentrations in influent and effluent; (**b**) COD removal rates; (**c**) stable COD concentration in influent and effluent and stable removal efficiency; (**d**) BOD concentrations in influent and effluent; (**e**) BOD removal rates; (**f**) stable BOD concentration in influent and effluent and stable removal efficiency; (**g**) SS concentrations in influent and effluent; (**h**) SS removal rates; (**i**) stable SS concentration in influent and effluent and stable removal efficiency.

The GDMBR system’s efficacy in removing organic contaminants and suspended particulate matters was comprehensively investigated and characterized through measurements of COD, BOD, and SS. The concentration of COD in fruit juice wastewater exhibited significant fluctuations during the 60-day continuous operation, as depicted in Figure 3a. The values ranged from 399.48 mg L^−1^ to 869.75 mg L^−1^, with an average influent COD value of approximately 666.83 mg L^−1^. After 60 days of filtration, the GDMBR demonstrated efficient COD removal, with an average COD concentration of 102.50 mg L^−1^ in the membrane effluent. It corresponded to a remarkable average removal rate of 82.99% (Figure 3b,c), indicating a significant achievement in COD removal efficiency by the GDMBR system. Moreover, the higher the concentration of COD, the greater the efficiency achieved in removing the COD. This phenomenon can be attributed to the synergistic effects of the sludge biodegradation and biofilm, collectively enhancing the removal of organic contaminants [40], as higher concentrations of organic matter promoted microbial growth and enhanced biological activity during the filtration process [10,41]. Meanwhile, the membrane permeate maintained a stable COD concentration despite fluctuations in the feed water during the filtration process. This observation suggested that the GDMBR exhibited robust resilience against variations in the COD concentration within the influent. It is worth noting that the average COD removal rate of the GDMBR exhibited a significant increase during the period from 25 to 60 days, compared to the preceding 25 days, which can be credited to the exceptional biodegradation capabilities of highly active sludge particles and mature biofilm during long-term filtration. Additionally, the BOD removal performance of the GDMBR system was assessed, and the findings are presented in Figure 3b–d. The concentration of BOD in the fruit juice wastewater exhibited a fluctuation range between 160.71 and 460.58 mg L^−1^, with an average value of 308.72 mg L^−1^. During the initial filtration period (1–10 days), a significant fluctuation in the influent water’s BOD concentration was observed, while the membrane permeate demonstrated an impressive average removal efficiency of 87.56%. Throughout the 11–60 day filtration period, a consistent stability was observed in the BOD concentration of the GDMBR’s membrane effluent, regardless of variations in the feed water’s BOD levels. This finding implied that an extended hydraulic retention time (HRT) progressively improves the biodegradation efficacy within the GDMBR system [42], thus strengthening the overall BOD removal. The average BOD concentrations in the effluent of the GDMBR were measured to be 30.85 mg L^−1^, with an associated average BOD removal efficiency of 89.95%. These findings suggest that employing a low-pressure membrane bioreactor can confer advantages in terms of enhancing resistance to shock loads of elevated BOD concentrations in the feed water and improving the overall BOD removal efficiency. The removal efficiency of suspended solids (SS) during the 60-day continuous filtration process in the GDMBR system is illustrated in Figure 3g–i. On day 1, the concentration of SS in the GDMBR was measured at 191.95 mg L^−1^, resulting in a remarkable removal rate of 84.92%. Subsequently, with the extension of filtration (1–10 days), there was an increase in microbial colonization within the active sludge and biofilm formation on the membrane surface, resulting in the gradual development of a cake layer. Consequently, following this period, a relatively stable trend was observed regarding SS removal with an impressive efficiency rate reaching 87.42% for the GDMBR. The inference can be drawn that, irrespective of variations in operational periods, the GDMBR consistently demonstrates comparable high-performance SS removal capabilities.

#### 3.2.2. Removals of Ammonia, Total Nitrogen, and Total Phosphorus

**Figure 4:**
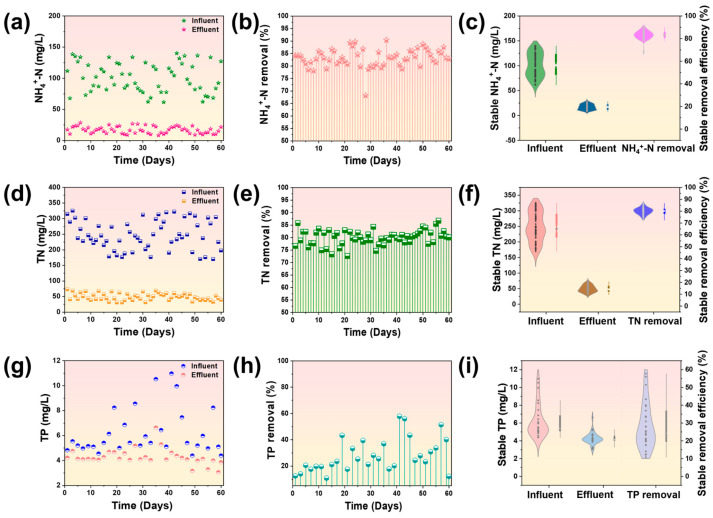
(**a**) NH_4_^+^-N concentrations in influent and effluent; (**b**) NH_4_^+^-N removal rates; (**c**) stable NH_4_^+^-N concentration in influent and effluent and stable removal efficiency; (**d**) TN concentrations in influent and effluent; (**e**) TN removal rates; (**f**) stable TN concentration in influent and effluent and stable removal efficiency; (**g**) TP concentrations in influent and effluent; (**h**) TP removal rates; and (**i**) stable TP concentration in influent and effluent and stable removal efficiency.

The ammonia removal performance is illustrated in Figure 4a–c. Throughout the entire experiment, the fruit juice wastewater exhibited an ammonia concentration ranging from 61.71 to 140.18 mg L^−1^. Remarkable reductions in the ammonia concentration were achieved in the membrane effluent of the GDMBR after filtration. The effluent demonstrated an average reduction in ammonia concentrations to 17.87 mg L^−1^, accompanied by an average removal rate of 82.47%. Moreover, it was observed that the GDMBR system maintained stable removal efficiency throughout the filtration process, indicating a minimal impact on performance from variations in the ammonia concentration. This phenomenon can be attributed to the successful cultivation of nitrifying bacteria within 25 days in the GDMBR system [10,43], which plays a pivotal role in achieving stable and efficient ammonia nitrogen removal.

The fruit juice wastewater exhibited a high concentration of nitrogen resources, which are indispensable for irrigation purposes, particularly in rural areas. Therefore, the TN concentration in the GDMBR system was investigated and is presented in Figure 3d–f. The fruit juice wastewater displayed fluctuating TN values ranging from 170.21 to 325.41 mg L^−1^. Following filtration by the GDMBR system, an average removal rate of 80.68% for the TN was observed, while the TN concentration in the membrane permeate of the GDMBR process showed significant fluctuations corresponding to changes in the initial water’s TN concentration. The removal of TN from the fruit juice wastewater was primarily achieved through the conversion to nitrogen by ammonia-oxidizing bacteria, nitrifying bacteria, and denitrifying bacteria [44].

The sustained presence of phosphorus in the membrane permeate of the GDMBR process is also crucial for irrigation, as it serves as another essential element. The removal performance of TP in the GDMBR system is illustrated in Figure 4g–i. The concentration of TP in the fruit juice wastewater ranged from 4.39 to 10.98 mg L^−1^. Over the initial 15 days of filtration using the GDMBR system, a notable TP removal efficiency was observed, with an average removal rate of approximately 22%. This can be attributed to adsorption and microbial uptake for metabolism and growth [45]. During the 7–13 day filtration period, the TP concentration in the effluent of the GDMBR closely approximated that in the influent water, with a slight removal rate of approximately 13% attributed to suspended sludge discharge. The fluctuations in the removal rates for phosphorus can potentially be attributed to the absence of sludge discharge during the 60-day operation, thereby hindering the GDMBR process from achieving optimal TP removal. As a result, TP could be mostly retained in effluent. Nitrogen (N) and phosphorus (P) are essential nutrients for crop growth, and they also offer potential value in treating fruit juice wastewater for irrigation purposes and subsequent phosphorus recovery in water [46,47]. The utilization of these macronutrients can enhance crop yields and further diminishes the reliance on mineral fertilizers, thereby mitigating environmental impact [48]. However, excessive concentrations of nitrogen and phosphorus can also result in incomplete crop assimilation and give rise to environmental issues such as eutrophication or groundwater contamination [49]. In this scenario, effectively irrigating with the membrane permeate from GDMBR based on crop growth requirements posed another noteworthy challenge.

### 3.3. Activated Sludge Properties

**Figure 5:**
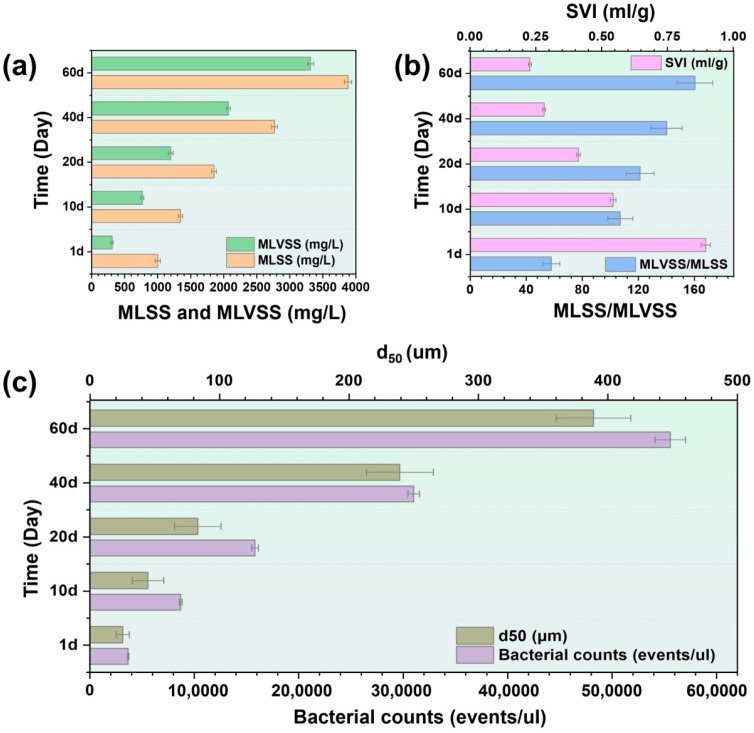
The sludge characteristics in the reactor: (**a**) MLVSS and MLSS; (**b**) SVI and MLVSS/MLSS; (**c**) *d_50_* and bacteria counts in the reactor.

The sludge samples were collected from the GDMBR reactor at 10-day intervals to investigate the dynamic variations in activated sludge properties and their impact on effluent performance (Figure 5a–c). Mixed liquor suspended solids (MLSSs) refers to the concentration of suspended solid particles in a system, while mixed liquor volatile suspended solids (MLVSSs) refers to the concentration of organic solid particles in the activated sludge of a system. The initial concentrations of MLSSs and MLVSSs in the reactor were 1002.39 mg L^−1^ and 309.62 mg L^−1^, respectively. By the end of the experiment, these concentrations increased to 3882.93 mg L^−1^ for MLSSs and 3317.03 mg L^−1^ for MLVSSs, indicating a tenfold rise in the organic solid substance concentration within the activated sludge of the mixed liquid after long-term operation. MLVSS/MLSS reflected the activity and biomass of activated sludge, which was increased by about 3-fold after long-term operation. The specific volume index (SVI) represents the volume of 1 g of dry sludge in the wet state, which is an indicator for assessing the settling performance of activated sludge. There was a decrease in the SVI value, from 168.09 mL g^−1^ to 42.62 mL g^−1^, signifying a significant enhancement in the sedimentation properties of the activated sludge during the process, thereby positively influencing the mitigation of sludge bulking [9]. Accordingly, as the bacterial counts in the system gradually increased from 3.6 × 10^4^ events μL^−1^ to 5.6 × 10^5^ events μL^−1^, it demonstrated remarkable biodegradability, ensuring the superior quality of the permeate. Furthermore, there was a gradual transition observed in the GDMBR system from floc-activated sludge to granule-activated sludge, as evidenced by an increase in the average particle size of sludge granules from 25.3 μm to 388.83 μm. Therefore, the GDMBR was conducive to biomass growth and aerobic granular sludge formation, which holds great potential for fruit juice wastewater treatment [50].

### 3.4. Microbial Community Analysis

#### 3.4.1. The Microbial Community Diversities

**Figure 6:**
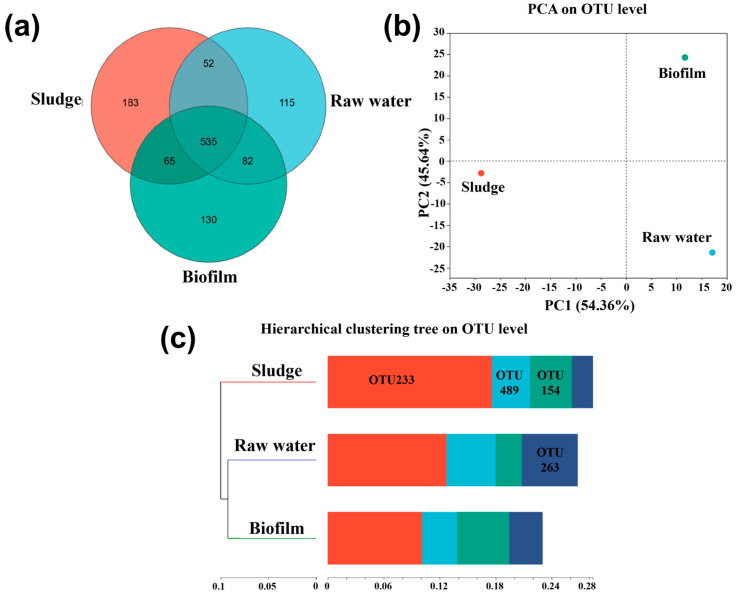
(**a**) Venn diagram showing unique and shared operational taxonomic units (OTUs) among the three sampling sites. (**b**) Principal component analysis (PCA) of the samples and (**c**) the OTUs number shift detected from samples.

By employing 16S rRNA technology, we aimed to unravel the intricate dynamics of microbial behaviors during sludge and biofilm development, thereby facilitating a comprehensive understanding of the biodegradability within the GDMBR system. The dissimilarity among microbial communities from the tested samples was visualized using a Venn diagram (Figure 6a), which shows the OTU numbers of shared and unique microbes among different treatments. A total of 535 OTUs were found to be shared across three samples. It was noteworthy that both the sludge and biofilm increased the number of unique OTUs compared to the raw water. Additionally, principal component analysis (PCA) was conducted to elucidate the relationship of microbial diversity among raw water, sludge particles, and biofilms. The sludge cluster was observed to be located in a distinct region, while the biofilm clusters and raw water were found to be adjacent to each other in a neighboring region (Figure 6b). This indicates that there were significant changes in the community structure between the sludge and raw water samples, but there was little difference between the raw water and biofilm. In the GDMBR system, the bacterial communities of the biofilm and raw water exhibited similarities; however, significant differences were observed in the bacterial communities between raw water and sludge, suggesting a relatively weak influence of the sludge community structure on biofilm growth. This phenomenon can be attributed to the ability of sludge particles to create a distinct microenvironment that modulates the impact of pollutants on microbial communities, resulting in divergent microbial communities in sludge compared to those found in both raw water and biofilm. On the one hand, the presence of carbon, nitrogen, and phosphorus adsorbed and encapsulated within sludge particles can serve as a valuable nutrient source for microbes, thereby augmenting their metabolic activity and potentially enhancing their capacity to degrade pollutants [51]. Furthermore, the OTU of bacteria in sludge was significantly higher than that in raw water, as demonstrated by the results presented in Figure 6c, suggesting that the progressive alternation of aerobic and anaerobic environments both inside and outside sludge particles can also facilitate the appreciation of specific microbial populations. These bacteria may have their own enhanced tolerance or degradation capabilities towards pollutants [52], thereby leading to the enrichment of microbiota diversity within sludge samples relative to raw water and biofilm.

#### 3.4.2. Key Functional Bacteria Involved in GDMBR Performance

**Figure 7:**
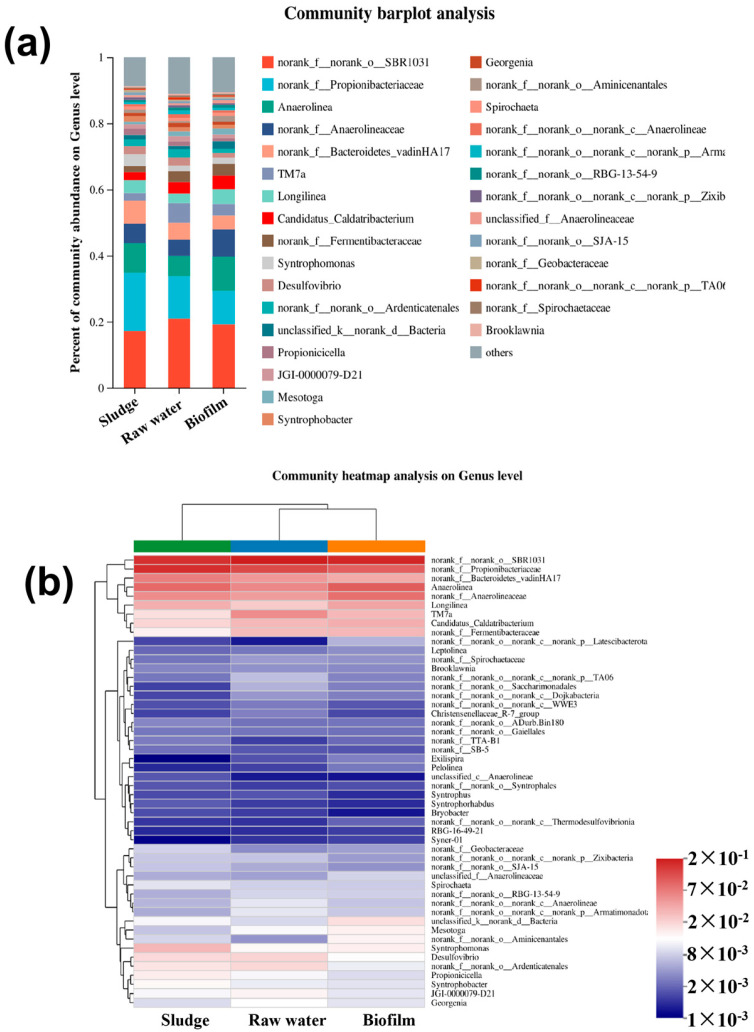
(**a**) Taxonomic classification of the bacterial communities in the GDMBR at genus levels; and (**b**) cluster analysis at genus levels.

The microbial community compositions were further analyzed and variations among the different samples at genus levels (with a relative abundance > 0.1%) were observed, as illustrated in Figure 7a,b. The results show that in the GDMBR system, the microbial communities of the raw water, sludge particles, and biofilm were similar, but the dominant species were different in magnitude. The predominant microbial communities identified were SBR1031 and Propionibacteriaceae, with SBR1031 accounting for 22.33%, 17.14%, and 19.45% of the total microbial population in the raw water, sludge, and biofilm samples, respectively. Meanwhile, Propionibacteriaceae accounted for 13.37%, 18.89%, and 11.13% in the corresponding samples. The secondary dominant microbial communities identified included Anaerolinea, Anaerolineaceae, and Bacteroidetes-vadinHA17 in the raw water, sludge, and biofilm samples, respectively. Among these samples, Anaerolinea constituted 6.33%, 11.23%, and 14.36% of the total microbial population, respectively, whereas Anaerolineaceae accounted for 4.17%, 7.12%, and 10.89%. Similarly, Bacteroidetes-vadinHA17 represented a proportion of 4.32%, 9.89%, and 2.43%. The bacterial populations were further assessed through phylogenetic analysis. Red indicates the predominant abundance of bacterial communities in each sample, while blue represents the inverse. Clearly, the abundance of Propionibacteriaceae, Anaerolinea, Anaerolineaceae, and Bacteroidetes-vadinHA17 exhibited a significant increase in the sludge samples compared to the raw water; however, only *Anaerolinea* and *Anaerolineaceae* showed an increase in the biofilm sample. *Propionibacteriaceae*, known as glucose fermenters, anaerobically produce propionic acid, acetic acid, succinic acid, and lactic acid. The enrichment of *Propionibacteriaceae* can provide small-molecule carbon sources to support microbial metabolism [53]. *Anaerolinea*, a saccharolytic bacterium, exhibited the capability of carbohydrate degradation, potentially contributing to glucose utilization for the production of intermediate metabolites in bioreactions [54]. The *Anaerolineaceae* family plays a pivotal role in the degradation of complex organic matter, thereby facilitating the initial stages of anaerobic digestion [55]. The *Bacteroidetes-vadinHA17* strain can facilitate the degradation of complex organic compounds through hydrolysis, thereby enhancing biodegradability [56]. These findings presented herein demonstrate a significant potential for the bioremediation of organic carbon/nitrogen contaminants in bioreactors, thereby substantiating the remarkable removal efficiencies exhibited by the GDMBR system towards organic and nitrogenous pollutants.

#### 3.4.3. Molecular Functions of Bacterial Microbiota in Systems

**Figure 8:**
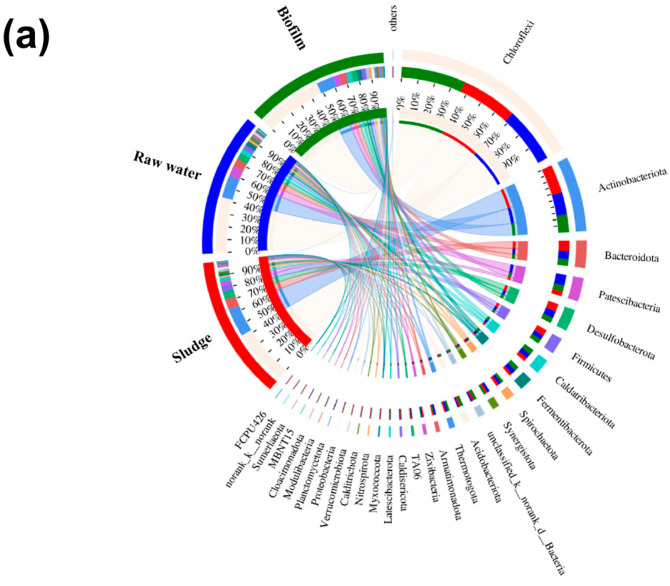

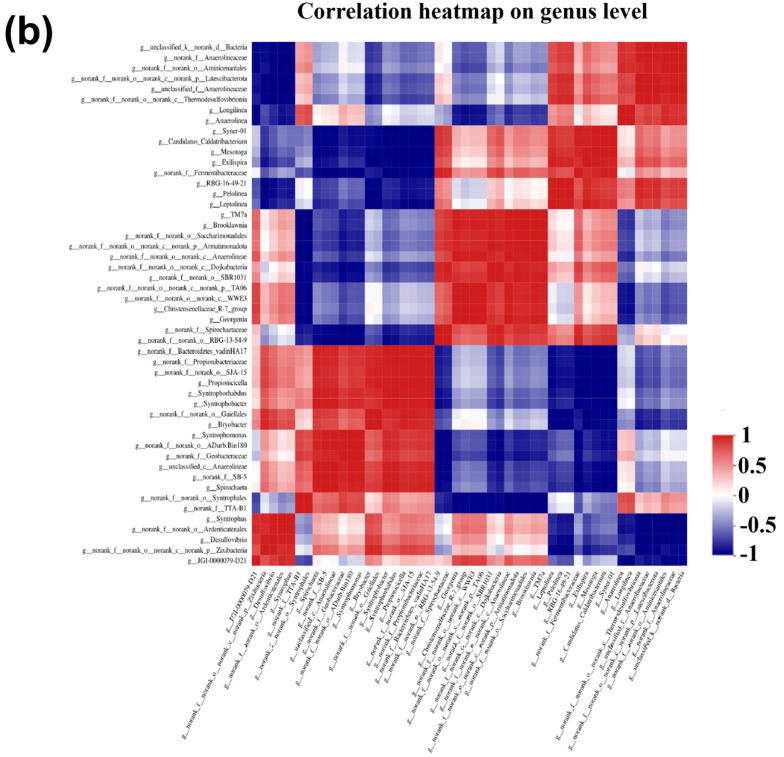
(**a**) Circos sample–species diagram; and (**b**) single-factor species correlation network heat map analysis.

The distribution of microbial species across diverse microbial samples was visualized using a Circos sample–species diagram (Figure 8a), and the interdependence between species was characterized through a single-factor species correlation network heat map analysis (Figure 8b). The color red represented the presence of symbiotic relationships among bacterial communities in the sample, whereas blue indicates their absence. The abundance of the *Chloroflexi*, *Actinobacteriota*, *Bacteroidota*, *Desulfobacterota*, and *Firmicutes* species in the sludge samples exhibited a significant increase compared to the raw water sample. Furthermore, the microbial composition of the biofilm closely resembled that of the raw water, exhibiting limited correlation with the biological species present in the sludge, which further provided the evidence for its origin from raw water. *Chloroflexi* exhibited diverse functionalities contingent upon environmental conditions, including their participation in organic degradation, nitrogen removal, and biofilm aggregation [57]. *Bacteroidota* and *Actinobacteriota* were found to exhibit a propensity for the production of humic acid (HA). These taxa significantly contributed to the degradation of organic matter transformation and nutrient cycling [58]. *Desulfobacterota*, a specie of sulfate-reducing bacteria, can actively participate in the anaerobic sulfate cycle [59]. *Firmicutes* and *Bacteroidota*, as autotrophic bacteria belonging to a group of anaerobic ammonium-oxidizing bacteria involved in partial nitrification and anaerobic ammonium oxidation processes, are efficient in total nitrogen removal without consuming organic carbon sources [60]. A single-factor species correlation analysis further revealed both symbiotic and mutually exclusive relationships among microbial species (Figure 8b). Specifically, as illustrated in the figure, the species residing within the red region exhibited a mutualistic association, enhancing each other’s biological metabolic capacity. Conversely, the species within the blue area were confined to the interactions that even constrained each other’s production metabolic capacity. Consequently, the GDMBR system accomplished an intrinsic integration of diverse microbial conversion pathways to augment the elimination of organic carbon and nitrogen pollutants, thereby showcasing its potential for efficient remediation.

## 4. Conclusions

The GDMBR process was initially employed in this study to directly treat real fruit juice wastewater without any pre-treatment procedures, with the aim of systematically evaluating its removal performance and flux level. Consequently, the following conclusions can be inferred.

(1) The GDMBR system exhibited flux stabilization regardless of variations in pressure levels, with the stabilized flux level consistently ranging from 2.5 to 4 L m^−2^ h^−1^. Furthermore, the stable flux achieved under 0.01 MPa in the GDMBR surpassed that obtained under 0.04 MPa.
(2) The GDMBR systems exhibited high microbial activity, and thus all GDMBRs demonstrated exceptional pollutant removal performance with average rates of 82.99% for COD, 87.56% for BOD, 82.47% for NH_4_^+^-N, and 80.68% for TN. Moreover, the GDMBR process effectively retained TP in the effluent to facilitate subsequent irrigation.
(3) The microbial communities in the raw water and biofilm of the GDMBR system exhibited similarities, but were notably distinct from those found in sludge, particularly in terms of dominant species. This suggests that the efficient removal of organic carbon and nitrogen pollutants primarily relied on the biological metabolic capacity of microorganisms in the sludge, while membrane pollution mainly originated from microorganisms in the raw water.

## Data Availability

The original contributions presented in this study are included in the article/Supplementary Material. Further inquiries can be directed to the corresponding authors.

## Supplementary Materials

The following supporting information can be downloaded at: https://www.mdpi.com/article/10.3390/membranes14120260/s1, Text S1: The membrane fouling resistance; Text S2: Water Quality Analysis.
