# Supplementary material for: A Gravity-Driven Membrane Bioreactor in Treating Real Fruit Juice Wastewater: Response Relationship Between Filtration Behavior and Microbial Community Evolution
Source: Membranes (Basel). 2024 Dec 6;14(12):260. doi: 10.3390/membranes14120260 (PMC11678127; doi:10.3390/membranes14120260)
Supplement: Supplementary file 1 [file membranes-14-00260-s001.zip › membranes-3339020-supplementary.pdf]

A gravity-driven membrane bioreactor in treating the real fruit juice wastewater: Response relationship between filtration behavior and microbial community evolution

Membranes

Dan Song[a], Haiyao Du[a], Shichun Chen[e], Xiaodie Han[a], Lu Wang[a], Gangyong Li[b,*], Caihong Liu[c,*], Wenjuan Zhang[d,*], Jun Ma[a]

[a]*School of Marine Science and Technology, Harbin Institute of Technology at Weihai, Weihai 264209, China*

[b]*Guangxi Key Laboratory of Urban Water Environment, Baise University, Baise 533000, China*

[c]*Key Laboratory of Eco-environments in Three Gorges Reservoir Region, Ministry of Education, College of Environment and Ecology, Chongqing University, Chongqing, 400044, China*

[d]*Tianjin Key Laboratory of Aquatic Science and Technology, School of Environmental and Municipal Engineering, Tianjin Chengjian University, Tianjin, 300384, P. R. China*

[e]*PetroChina Harbin Petrochemical Company, Harbin 150056, China*

**corresponding authors:*

E-mail: caihong.liu@cqu.edu.cn, wenjuanvivian@126.com

Text S1.

Total membrane resistance (R_t, m^{-1}) can be divided into membrane inherent resistance (R_m, m^{-1}) and fouling resistance (R_f, m^{-1}). R_f was composed of reversible fouling resistance (R_r, m^{-1}) and irreversible fouling resistance (R_{ir}, m^{-1}). Filtration membranes were placed in the systems and run using pure water to obtain initial membrane permeability (J_0, L/m^2h). R_m is calculated as Eq. (S1). The final membrane permeability was denoted as J_1 to calculate R_t as shown in Eq. (S2). Then, the fouling layer of the membrane was wiped off by hydraulic flushing, and the membrane was filtrated again using pure water. The stable permeability of the membrane was recorded as J_2. R_r and R_{ir} are calculated as Eq. (S3) and Eq. (S4), respectively. ΔP (Pa) is the transmembrane pressure, μ (Pa·s) is the viscosity coefficient of water at 25±1 °C.

$$R_m = \frac{\Delta P}{\mu J_0} \tag{S1}$$

$$R_t = \frac{\Delta P}{\mu J_1} \tag{S2}$$

$$R_{ir} = \frac{\Delta P}{\mu J_2} - \frac{\Delta P}{\mu J_0} \tag{S3}$$

$$R_r = \frac{\Delta P}{\mu J_1} - \frac{\Delta P}{\mu J_2} \tag{S4}$$

Text S2.

Water Quality Analysis:

Chemical oxygen demand (COD) was examined by traditional procedure in which 100 mL of water sample was homogeneously mixed with 5 mL of H_2SO_4 (1:3) solution and 10 mL of 0.01 mol L^{-1} $KMnO_4$ solution. The mixture was put into water bath at 100 ºC for 30 min to carry out the oxidation reaction. After digestion, water sample was inoculated with 10 mL of 0.01 mol L^{-1} $Na_2C_2O_4$ and titrated by the as-prepared $KMnO_4$ solution. Ammonia nitrogen (NH_4^+-N) concentration was measured by Nessler's reagent spectrophotometry. BOD was measured according to national standard. A standardized approach for the determination of total phosphorus using the ammonium molybdate spectrophotometric method.
